# Adaptive assistive robotics: a framework for triadic collaboration between humans and robots

**DOI:** 10.1098/rsos.221617

**Published:** 2023-06-28

**Authors:** Daniel F. N. Gordon, Andreas Christou, Theodoros Stouraitis, Michael Gienger, Sethu Vijayakumar

**Affiliations:** ^1^ The University of Edinburgh, Edinburgh, UK; ^2^ The Alan Turing Institute, London, UK; ^3^ Honda Research Institute Europe, Offenbach, Germany

**Keywords:** ergonomics, optimization, optimal control

## Abstract

Robots and other assistive technologies have a huge potential to help society in domains ranging from factory work to healthcare. However, safe and effective control of robotic agents in these environments is complex, especially when it involves close interactions and multiple actors. We propose an effective framework for optimizing the behaviour of robots and complementary assistive technologies in systems comprising a mix of human and technological agents with numerous high-level goals. The framework uses a combination of detailed biomechanical modelling and weighted multi-objective optimization to allow for the fine tuning of robot behaviours depending on the specification of the task at hand. We illustrate our framework via two case studies across assisted living and rehabilitation scenarios, and conduct simulations and experiments of triadic collaboration in practice. Our results indicate a marked benefit to the triadic approach, showing the potential to improve outcome measures for human agents in robot-assisted tasks.

## 1. Triadic collaboration

Human–robot collaboration involves the cooperation between human and robotic agents, in order to achieve shared goals. Unlike in traditional industrial robotics environments, where robotic agents are often physically separated from human workers via barriers to prioritize safety [1], collaborative robots (often termed *cobots* [2,3]) exploit direct physical interaction between humans and robots to assist with complex or physically demanding tasks [4].

**Figure 1.**
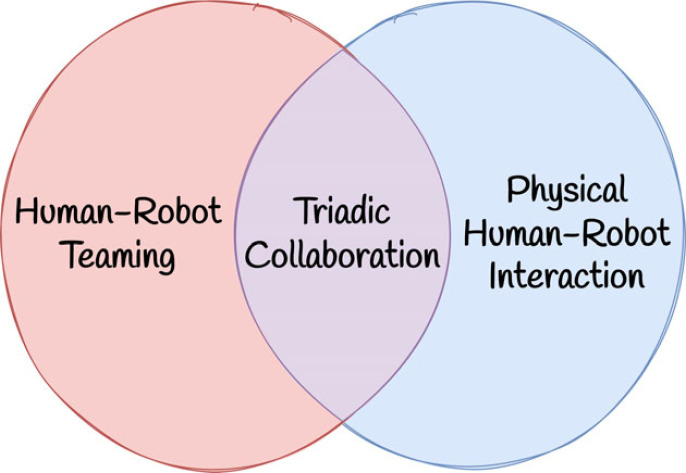
Triadic collaboration lies at the intersection of human–robot teaming and pHRI.

Human–robot collaboration scenarios can in some sense be characterized on a spectrum of physical interaction and number of agents. On one extreme, human–robot teaming involves cooperation between multiple humans and (potentially large numbers of) autonomous robotic agents [5,6]. Physical human–robot interaction (pHRI) scenarios typically involve collaboration between a single human and robot, with a heavy focus on the nature of the direct physical interaction between the two [7]. The intersection of human–robot teaming and pHRI comprises problems which involve multiple human and robotic agents, with physical interaction between some subset of the agents. We describe these scenarios as *triadic collaboration* problems (figure 1).

Triadic collaboration scenarios are increasingly becoming ubiquitous throughout many industries and domains ranging from factory-line work to rehabilitation centres. This is driven by the increasing levels of adoption of robot and exoskeleton technologies in the workplace [8], where they are used to increase productivity and reduce the physical stress on staff and healthcare professionals. Those who engage in labour-intensive or physically repetitive tasks in the workplace have been shown to be at a higher risk of developing musculoskeletal disorders (MSDs) over the course of their life [9–11]. Examples of such occurrences are numerous, ranging from nurses developing back injuries due to lifting and otherwise assisting patients [12], to office workers being susceptible to neck pain and injury due to ergonomically unsafe working postures [13]. MSDs cause a loss of productivity [14], and can have significant negative physical and psychological effects on workers [15].

Concrete examples of triadic collaboration include:

(i) The introduction of a robotic workforce to augment and assist an existing human staff [16]. In this scenario, human and robotic agents share the same physical space and collaborate to achieve shared goals [17,18], while wearable robotic devices can be worn by workers to directly provide assistive torques to the human joints, with the high-level aim of minimizing ergonomic risk [19,20].
(ii) The assisted living and wider healthcare settings. While research in this area is in its early phase, exoskeletons are thought to have great potential to assist healthcare professionals like nurses in their daily tasks while reducing risk of injury [21,22].
(iii) In physiotherapy, where exoskeletons and other assistive technologies have shown potential as a tool for improving rehabilitation outcomes [23,24].

Notably, these examples of triadic collaboration take place in various application domains and on the surface appear to be entirely disparate problems, with scenarios differing in both the composition of agents, as well as the nature of the physical interaction between agents. For instance, in scenario (i), the composition of agents is mixed between humans and robots, whereas scenario (iii) features a single human agent interacting with multiple technologies. Despite these differences, these examples of triadic collaboration do share a set of characteristic features:

— one (or more) human agents collaborating with one (or more) robotic agents,
— physical interaction between at least some agents,
— a set of high-level common goals.

We propose to encapsulate each of these examples, and more, within a generic framework for tackling triadic collaboration scenarios, which we define as those exhibiting the three characteristic features listed above.

## 2. A framework for triadic collaboration problems

**Figure 2.**
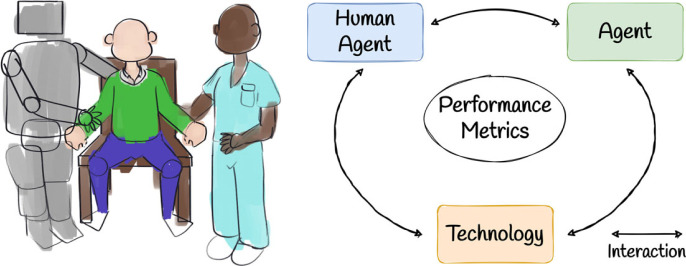
A general illustration of triadic collaboration. In the general case, this comprises a mixture of agents, of which at least one is human and at least one is technological, with some level of physical interaction between agents. An example, as depicted, is a nurse and robot collaborating to help a patient perform a sit-to-stand.

The high-level aim of our framework is to determine the most optimal behaviours for robotic agents in triadic collaboration scenarios (figure 2). When carrying out a triadic collaboration task, typically there are one or more high-level objectives which are to be realized. For example, for a factory worker engaging in a repetitive overhead task, an active assistive exoskeleton should be controlled to assist in completion of the task, while also minimizing the risk of shoulder injury to the worker. The level of ergonomic risk associated with a task, as well as the methodology for quantifying that risk, is highly dependent on the task at hand. Therefore, our triadic collaboration framework has two key requirements:

(i) *the ability to carry out motion tasks while minimizing ergonomic risk for human agents*,
(ii) *the ability to generalize to various triadic collaboration scenarios*.

A natural setting which allows us to achieve these requirements is that of mathematical optimization. More concretely, we consider an optimization problem whereby our objective function consists of a weighted sum of *ergonomics metrics*, which describes the level of ergonomic risk associated with specific tasks (box 1).

Box 1. **Optimization framework.**

Our generic formulation of the triadic collaboration problem is as follows:

2.1 $$\begin{array}{rl} & \min_{x}\;\sum_{k=1}^{K}w_{k}E_{k}(x),\\ & s.t.\;M_{i}(x)\geq 0,\;M_{\;j}(x)=0,\;\text{for}\;M\in M\\ & \;\;\;T_{i}(x)\geq 0,\;T_{\;j}(x)=0,\;\text{for}\;T\in T. \end{array}$$

Here, $x\in R^{n}$ are the *optimization variables*, which represent decision variables for the combined set of human and robotic agents, and $E_{k}\;:\;R^{n}\mapsto R$ are *ergonomics metrics*, which together with associated *weights* $w_{k}\in R$ define the *objective function* as a summation of *K* *weighted ergonomics metrics*. In addition, our problem contains two categories of constraint: *task constraints* T∈T, which codify the constraints which are required to ensure task completion, and *modelling constraints* M∈M, which represent system dynamics. These constraints are represented as a combination of inequality constraints (*M*_*i*_, *T*_*i*_) and equality constraints (*M*_*j*_, *T*_*j*_).

Our framework uses biomechanical models which introduce additional computational complexity in return for a much more detailed appreciation of these ergonomics metrics, as we will see in the next section.

### 2.1. Quantifying ergonomics via biomechanical modelling

As discussed previously, the use of exoskeletons and other robotic agents in the workplace is driven, at least in part, by the desire to reduce the impact of MSDs in the workplace. *Ergonomics metrics* provide a means of quantifying the level of risk of experiencing an MSD associated with certain motions or tasks—and therefore are prime candidates for optimization via our triadic collaboration framework.

A well known and widely used ergonomics metric is the *rapid entire body assessment* (REBA) [25]. The REBA metric assigns a score of 1–15 for a task according to the perceived ergonomic risk. The overall score is dependent on multiple factors, including the effects of heavy loads and strenuous activities, but is largely dependent on a kinematic analysis of various parts of the body such as the neck, torso and legs. Other ergonomics measures have been developed [26] which typically share many features with REBA—namely, a consideration of largely kinematic features (e.g neck angle, torso angle) with a relatively coarse consideration of dynamic effects (i.e load carried), and a low temporal resolution, whereby ergonomics scores are generated only for complete tasks or after observing a task for some fixed amount of time.

**Figure 3.**
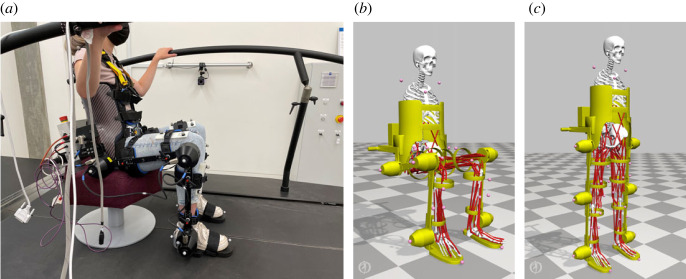
(*a*) A research participant wearing a lower-body exoskeleton, taking part in an investigation of sit-to-stand biomechanics. (*b*,*c*) Snapshots from a reconstruction of the sit-to-stand motion. Musculoskeletal models, built in OpenSim [30], account for the coupling between human and exoskeleton, and allow for detailed analysis of the behaviour of human joints and muscles.

More recently, researchers have employed the use of musculoskeletal models (figure 3) in the analysis of ergonomics [27–29]. These models represent the human body as a composition of bodies, joints and muscles—which together constitute the human’s body system dynamics. They can be personalized to match a particular subject via a process of model scaling and parameter specification, and can target a level of complexity appropriate to the task at hand.^1^ A powerful feature of these models is the ability to explicitly model the presence of muscle weakness or pathology via a direct adjustment of the appropriate muscle parameters. In addition, external devices like exoskeletons can be physically coupled to the human model (figure 3), thus directly impacting the system dynamics. Computing ergonomics measures via musculoskeletal modelling is inherently more computationally complex than existing, data-driven metrics like REBA. However, these approaches can offer numerous advantages, including a typically higher temporal resolution (i.e. the ability to quantify risk at given points during task execution as opposed to on a task-by-task basis), as well as the ability to consider the behaviour of muscles and forces in addition to kinematic trajectories [31–33]. This level of granularity can be important, particularly for triadic collaboration tasks that require a more careful consideration of ergonomics, e.g. when controlling an exoskeleton to avoid or reduce the risk of injury to a particular joint or set of muscle groups.

### 2.2. Optimizing the actions of robotic agents

Given appropriate biomechanical models and constraints *T* which describe a triadic collaboration task, we can use the optimization framework outlined in System (2.1) to optimize robot behaviour—this procedure is demonstrated further in the forthcoming case studies. There are two powerful modifications we can make to the framework on a case-by-case basis to fine-tune robot behaviour as needed:

(i) modifying the components (and relative weightings) of the objective function, to suit a large variety of triadic collaboration tasks, or account for differences between human agents,
(ii) explicit modifications to the dynamics models, which can represent injury or muscle pathologies in human agents.

**Figure 4.**
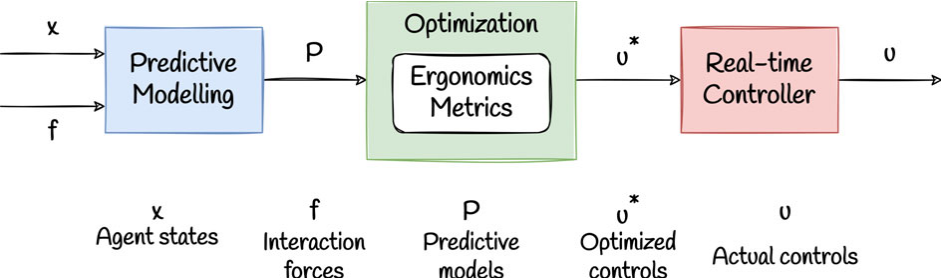
A schematic outlining the relationship between the key components of the triadic collaboration framework. This can be interpreted as a control diagram showing inputs to and outputs from each component of the framework at time *t*. The formulations discussed in this work refer to the optimization component, shown in green.

In practice, the triadic collaboration framework involves a composition of predictive modelling, optimization, and real-time control blocks as outlined in figure 4. The predictive modelling block enables *partner policy prediction*, i.e the ability for robot agents to predict how their actions will affect future actions of the human agents, and is an important component of human–robot collaboration frameworks in general [18]. The precise implementation of the predictive modelling and real-time control blocks are problem-specific.

## 3. Case study i: assisted living and working

**Figure 5.**
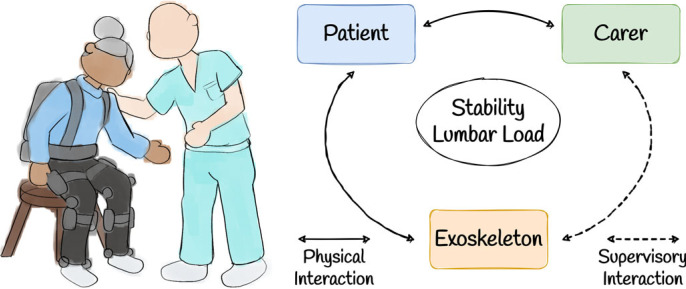
A schematic illustrating the triadic collaboration framework instantiated in an assisted living scenario. Here, a human agent such as a nurse or carer (the assistance provider) is providing physical assistance to a patient (the assistance seeker) with additional support from a technological agent (e.g. an exoskeleton). The assistance seeker has direct physical interaction with both the technology and the assistance provider, while the assistance provider interacts with the technology only in a supervisory fashion.

In this case study, we consider the example of a human patient being assisted in a sit-to-stand manoeuvre by a combination of a robotic exoskeleton and a human carer. More generally, we can consider this as a subcase of a more general scenario in which a human *assistance seeker* interacts with a human *assistance provider* and a form of *assistive technology* (figure 5). An additional subcase is briefly outlined in box 2. The assistive technology in these scenarios has a direct physical link to only the assistance seeker; therefore, the assistive technology can only affect the behaviour of the assistance provider by first interacting with the assistance seeker. This general specification exemplifies the use of assistive technology to assist multiple human agents.

### 3.1. Problem formulation

The primary objective of both the assistive technology and the assistance provider is to ensure the safety of the assistance seeker. A secondary but nevertheless important objective is to minimize the ergonomic risk of injury to the human carer. In our framework, these objectives can be achieved via insertion of appropriate ergonomics metrics into the objective function of our framework (system (2.1)):

— *margin of stability* of the assistance seeker, which describes fall risk,
— *lumbar joint loading* of the assistance provider, to reduce the risk of back injury.

Mathematically, given some motion which started at time *t* = 0 and ended at time *t* = *t*_*f*_, these ergonomics metrics can be written as follows:

stability $$E_{s}=\int_{0}^{t_{\;f}}c_{\;p}+\frac{c_{v}}{w_{0}}\;dt$$

and

lumbar loading $$E_{b}=\int_{0}^{t_{\;f}}\omega^{T}F_{b}\;dt,$$

where *c*_*p*_, *c*_*v*_ , respectively, denote the position and velocity of the centre of mass, *w*_0_ is a constant determined by leg length, **F*_b_*** is the six-dimensional vector of net forces and torques acting on the carer’s back, and ***ω*** is an internal weighting vector which controls the relative importance of each generalized force component. These metrics can be inserted into system (2.1) as follows:

3.1 $$\underset{x}{\min}\;w_{s}E_{s}+w_{b}E_{b},$$

where *w*_*s*_ and *w*_*b*_ encode the relative weighting of the ergonomics metrics.

Next, we consider how to model the action of exoskeleton and external agent assistance in the form of constraints for system (2.1). These take the following form:

dynamics $$M(q)\ddot{q}+c(q,\dot{q})+g(q)+F_{a}=\tau_{h}+\tau_{e}$$

and

exoskeleton assistance $$\tau_{e}=f_{e}(x).$$

Here, the components **F**_*a*_ and ***τ***_*e*_ represent the contributions of the assistive agent and the assistive technology, respectively, to the general equation of multi-body system dynamics [34]. The function *f*_*e*_(*x*) maps the generated exoskeleton motor torques on to the human body via an appropriate exoskeleton force transmission model [32], where exoskeleton commands are now included in the optimization variable vector *x*.

Finally, we include task constraints which encode the initial (sitting) and final (standing) configuration of the assistance seeker as follows:

task constraints $$\;\begin{array}{cc} & q(0)=q_{s}\\ \;\;\;\;\;\;\;\;\; & q(t_{\;f})=0, \end{array}\}$$

where **q**_*s*_ corresponds to a sitting pose.

Box 2. **Assisted working.**

**Figure 6.**
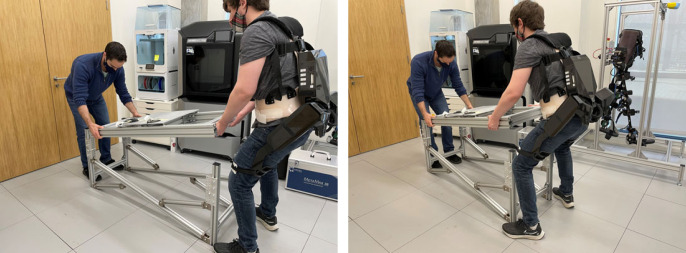
Snapshots of an example triadic collaboration work task involving two human agents and an exoskeleton. One human agent bears the brunt of the load, with the assistance of an exoskeleton providing ergonomic support. Meanwhile, the second human agent carries out the finer manipulation.

Already, state-of-the-art exoskeletons for ergonomics support are being employed in industrial settings [35]. However, these applications remain largely dyadic in nature i.e. one human worker being assisted by a robotic agent. The example instantiation of the triadic collaboration framework presented here could naturally be extended to the case of assisted working (figure 6), to unlock scenarios in which human agents and robots collaborate simultaneously. For example, the composition of stability and lumbar loading in the objective function could be directly applied to a manual lifting and carrying task.

### 3.2. Indicative results

**Figure 7.**
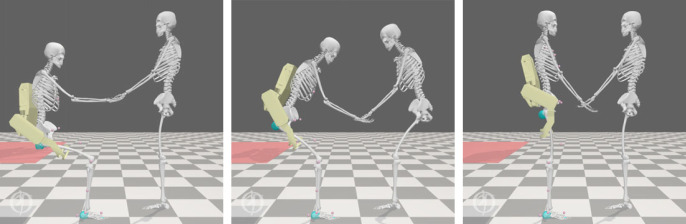
Snapshots of a simulated assisted sit-to-stand transition. A patient (left) is assisted in completing a sit-to-stand by a combination of a human carer (right) and an exoskeleton (highlighted in yellow). The red plane models a seat, while blue spheres represent contact geometries.

To evaluate the benefits of our formulation, we can leverage the use of digital twins to simulate triadic collaboration scenarios. The assisted sit-to-stand scenario is modelled using open-source musculoskeletal modelling software OpenSim [30] and shown for reference in figure 7. The human agents are represented by a two-dimensional musculoskeletal model with 6 dof representing the movements of the back, hip, knee, ankle, shoulder and elbow joints in the sagittal plane. The joints are actuated by torque actuators which feature activation dynamics. The feet of each model are constrained to the ground, with a geometric kinematic constraint used to link the hands of the human agents and allow the transfer of force during the sit-to-stand movement. The agent representing the assistance seeker has additional contact geometries to represent the initial sitting configuration. The robotic agent, shown in yellow in figure 7, is a computer-aided design (CAD)-based representation of the active pelvis orthosis (APO) exoskeleton [36], a powered mobile exoskeleton for movement assistance. In this model, it is represented by its mass properties as well as two ideal torque actuators located on the hip and back joints of the carer agent, which each have a peak torque of 150 Nm.^2^

The objectives and constraints outlined in the problem formulation are implemented in OpenSim Moco [37], an open-source software which uses direct collocation to solve optimal control problems with OpenSim models. A range of simulations were conducted to investigate both the impact of joint weakness on human sit-to-stand biomechanics, as well as the efficacy of an approach based on triadic collaboration.

The parameters varied between simulations included the exoskeleton assistance level, the strength of the musculoskeletal model representing the assistance seeker, and the form of the objective function used in the optimal control problem. In two simulations, exoskeleton assistance was disabled, so as to gain insight into dyadic human–human sit-to-stand strategies, while in the remaining simulations exoskeleton assistance was enabled. In one simulation, both human agents were at full strength—this represented a ‘healthy’ sit-to-stand, while in the remaining simulations the joint strengths of a subset of the assistance seeker’s joints were reduced by 90% in order to simulate muscle weakness. Finally, two simulations focused specifically on overweighting the stability and lumbar loading cost terms, respectively, so as to provide a comparison for the influence of triadic collaboration. The test-cases evaluated are summarized for reference in table 1.

**Table 1.** The experimental conditions for each sit-to-stand transition simulation. Note that the relative disparity between the baseline values of *w*_*b*_ and *w*_*s*_ (10^−4^ and 1, respectively) arises due to the difference in the typical order of magnitude of the corresponding cost terms.

simulation	assistance enabled	joints weakened	*w*_*b*_	*w*_*s*_
1	no	none	10^−4^	1
2	no	back, hip, knee	10^−4^	1
3	yes	back, hip, knee	10^−6^	1
4	yes	back, hip, knee	10^−4^	10^−2^
5	yes	back, hip, knee	10^−4^	1
6	yes	ankle	10^−4^	1

To enable a statistical consideration of the results, each simulation case was run for five simulated human subjects, corresponding to the assistance seeker in the scenario, which differed in their mass properties and joint strengths. Subject 1 was implemented with peak joint torques of 200 Nm and a total mass of 65.9 kg. The remaining subjects were randomly assigned a peak joint torque and mass within ±20% of these baseline values. The model representing the assistance provider was unchanged over the simulations. The system properties of the simulated subjects are summarized for reference in table 2.

**Table 2.** The system mass and joint strengths assigned to each of the simulated subjects corresponding to assistance seekers in the case study i simulations.

subject	mass (kg)	joint strength (Nm)
1	65.9	200
2	75.2	172
3	67.9	208
4	73.2	208
5	67.2	174

**Figure 8.**
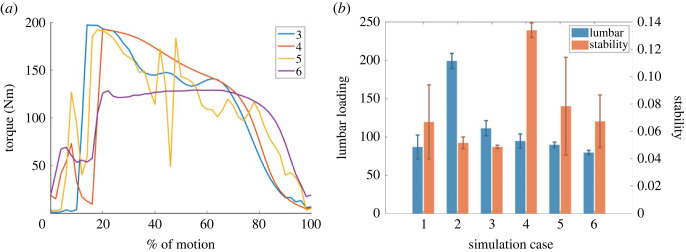
(*a*) The assistive torques generated by the APO in simulations 3–6. (*b*) The lumbar loading and stability costs for each simulated test case. Lower costs indicate better performance.

**Figure 9.**
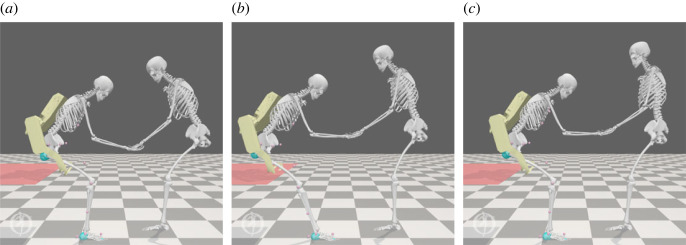
Intermediate snapshots from simulations 3 (*a*), 4 (*b*) and 5 (*c*) showing the agent configurations after 0.5 s of the sit-to-stand transfer. Note in particular the more acute angle of the assistance provider’s back joint in Simulation 3, in which lumbar loading is not optimized.

The results of the assisted sit-to-stand simulations are summarized in figure 8, and snapshots from simulations 3–5 are shown in figure 9 to illustrate the changes in biomechanics induced by modifications to the overall objective. Firstly, comparing the results of simulation 1 and simulation 2, we note that the weakening of the assistance seeker has had a significant effect on the overall sit-to-stand biomechanics, significantly increasing the lumbar loading metric. This is as expected, since without exoskeleton assistance the burden of making up for the lack of strength lies solely with the assistance provider, which places additional strain on the lumbar joint.

In simulations 3–6, where exoskeleton assistance is enabled, lumbar loading is markedly reduced. Simulations 3 and 4 sacrifice lumbar loading for stability, and vice versa, as expected due to the overweighted nature of the cost function in these cases. The equitable inclusion of the lumbar loading cost term in the objective function of simulation 5 results in a marked reduction of lumbar loading when compared with simulation 3, though a relative decrease in stability does occur in this case. Interestingly, the relative variance in the stability cost is significantly larger than that of the lumbar loading cost, and this is particularly true for certain simulations (1, 5 and 6), suggesting that changes in mass and model strength have a stronger effect on stability than lumbar loading of the carer. This is perhaps a natural consequence of the stability metric being largely based on the dynamics of the assistance receiver. Simulation 6 was included to investigate the effect of different musculoskeletal pathologies on sit-to-stand biomechanics, and differs from simulation 5 only in the joints weakened in the musculoskeletal model. The optimized APO assistance results in decreases to both the lumbar loading and stability costs.

The assistive forces generated by the APO in these simulations are shown for comparison purposes in figure 8. Notably, the peak in assisted torques occurs much earlier than observed in human sit-to-stand data [38]. The difficulty of hand-tuning such trajectories highlights the benefits of our optimization-based approach.

It is particularly notable that despite no direct physical link between the exoskeleton and the assistance provider, it is capable of reducing the physical strain experienced by this agent without overly compromising the stability of the assistance seeker (i.e. comparing the mean stability from simulation 1 with simulation 5). Alternative cost term weightings could be chosen based on the desired outcome of the assistance pattern, i.e. the weighting from simulation 3 or 4 depending on whether stability or lumbar loading are more important to the task at hand. This behaviour clearly motivates the treatment of exoskeleton control in such scenarios as a triadic collaboration problem; whereby multiple agent-specific objectives can be included and prioritized according to the nature of the motion task.

## 4. Case study ii: robot-assisted rehabilitation

**Figure 10.**
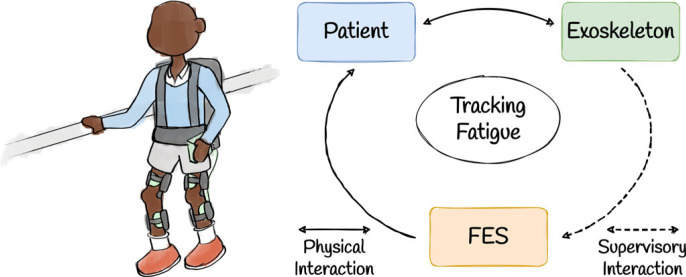
A schematic illustrating the triadic collaboration framework instantiated in a specific rehabilitation scenario. Here, a human agent carries out a prescribed motion with assistance from two technological agents—an exoskeleton and FES electrodes.

Here, we consider an instance of triadic collaboration involving two technological devices assisting a single human agent. A typical real-world example of this is in rehabilitation centres, where physiotherapists may use a combination of functional electrical stimulation (FES) and exoskeleton assistance to achieve a desired rehabilitation plan (figure 10). The assistive agents in this scenario share the goal of assisting the human to follow a kinematic trajectory; however, they do so with the additional objective of fatigue minimization; if muscle fatigue is low, more FES is used to encourage muscle strengthening, but if muscle fatigue is high, the exoskeleton picks up more slack to allow the human muscles to rest. Therefore, we see that the relative balance of FES and exoskeleton assistance is in a trade-off relationship with the level of muscle fatigue currently experienced by the assistance seeker.

### 4.1. Problem formulation

In our framework, the trade-off between muscle fatigue and assistance level is achieved via a combination of goals and constraints. The goals take the following form:

tracking $$E_{t}=\int_{0}^{t_{\;f}}{\left(q_{\mathrm{ref}}-q\right)}^{2}\;dt,$$

assistance $$E_{u}=\int_{0}^{t_{f}}\|u_{e}{\|}_{2}\;dt$$

fatigue $$\;E_{\;f}=\int_{0}^{t_{\;f}}f_{m}(q,\dot{q},P)\;dt,$$

where **q** contains the human joint trajectories, which are desired to track reference trajectories **q**_ref_, **u**_e_ represents exoskeleton torque commands, and *f*_*m*_ is an equation modelling muscle fatigue [39]. To represent the action of FES and exoskeleton assistance, we can introduce the modelling constraints from the previous case study, in addition to a further constraint,

FES $$\tau_{h}=\tau_{n}+f_{s}(x),$$

which indicates that the combined human joint torques ***τ***_*h*_ are now a composition of the natural contribution ***τ***_*n*_ and a FES-induced contribution modelled by the function *f*_*s*_. In this case, input commands to the FES electrodes, as well as exoskeleton motor commands, are both now included as optimization variables.

Box 3. **Assist-as-needed control.**

A key concept in the wider area of robot-assisted rehabilitation is *assist-as-needed* control, whereby robot control signals should only act to support humans when necessary, and otherwise should not affect human efforts. This arises as a natural consequence of the balancing of muscle fatigue and trajectory tracking in our optimization—if the human is already tracking the input trajectory well enough, and fatigue is low, no additional inputs are required from the technological agents. On the other hand, if tracking performance drifts, or measured fatigue becomes high, the assistive technology is able to pick up the slack. The ‘slack’ offered by the technological agents can be tuned on a person-specific basis by the physiotherapist by varying the relative magnitude of the weighting terms *w*_*t*_ and *w*_*f*_ associated with the tracking and fatigue costs, respectively.

The relative magnitude of the weighting between trajectory tracking, exoskeleton assistance, and muscle fatigue can be varied on a temporal basis as a patient’s condition progresses or improves during the rehabilitation process. For example, a patient recovering from a recent stroke could at first be assigned a high fatigue weighting which is gradually reduced as their strength improves. This ability to fine tune the precise behaviour of triadic collaboration protocols depending on individual requirements is a powerful feature of the optimization-based control framework we have presented here (see equation (2.1) in box 1).

### 4.2. Indicative results

**Figure 11.**
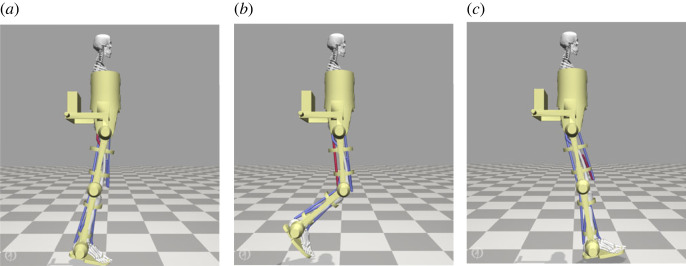
Snapshots of a simulated assisted swing leg motion. A patient is assisted in completing the swing leg motion by a combination of exoskeleton assistance and electrical stimulation. During the initial swing (*a*), the gluteus muscle is stimulated, during the mid-swing (*b*) the hamstring muscles are stimulated and during the terminal swing (*c*) the vasti muscles are stimulated. The stimulated muscles are presented in red and the non-stimulated muscles are presented in blue.

To illustrate the optimization of hybrid robot-FES control parameters via our triadic collaboration framework, we consider a simulation of an assisted trajectory tracking task (figure 11). In this case, the musculoskeletal model representing the human-agent contains 10 dof and is actuated by 18 muscle-based actuators. A CAD-based representation of the H3 exoskeleton (Technaid, Spain) is affixed to the model, and contains 6 active degrees of freedom across the hip, knee and ankle joints, each of which is powered by an ideal torque actuator. The human-exoskeleton interface is modelled via bushing forces, which act as six-dimensional spring-damper systems and represent the action of the exoskeleton straps.

The equation of motion for the combined system of agents is as follows:

4.1 $$M(q)\ddot{q}+c(q,\dot{q})+g(q)+F_{a}=\tau_{n}+\tau_{\;f}+\tau_{e}.$$

Compared with case study i, we have an additional term ***τ***_*f*_, which results from the presence of FES in the human component of the net torque vector, and the isolated human contribution is denoted as ***τ***_*n*_. A linear model is used to approximate the effect of electrical stimulation on human muscle activity

4.2 $$a_{c}=a_{h}+a_{\;f},$$

where the subscripts *c*, *h* and *f* denote the combined activity, the activity due to human intention and the activity due to FES, respectively—note that the combined activity of any muscle is constrained to lie within [0, 1]. This simple model assumes that electrical activity from the FES electrodes are perfectly transferred to the human neuromuscular system.

**Figure 12.**
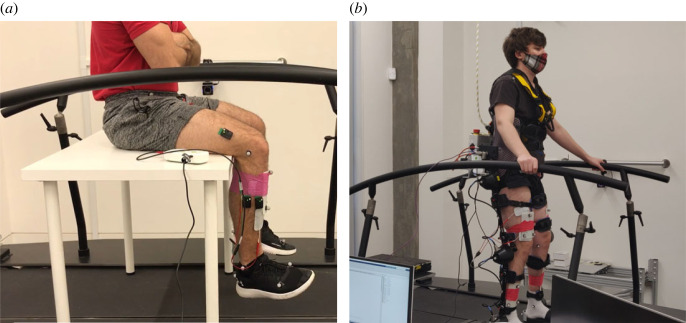
(*a*) A subject undergoing familiarization with the FES electrodes. (*b*) A subject undergoing the trajectory tracking task with the H3 in transparent mode, to obtain the baseline human intention.

The human intention, in the form of prescribed muscle activity **a**_*h*_, was approximated using motion capture and reconstruction [32] from subjects in the University of Edinburgh gait laboratory. The raw motion data were obtained from an experimental set-up in which subjects followed a prescribed tracking task while wearing the H3 exoskeleton in transparent mode [40]. Images of the experimental set-up, including FES electrodes which were deactivated for this initial data collection procedure, are shown in figure 12.

To estimate the muscle activation induced by FES, **a**_*f*_, a simplified muscle activation model is used [41]. A linear function is used to obtain the induced activation for a given muscle based on the stimulation pulse width **u**_*f*_, threshold **u**_thr_, and saturation pulse width **u**_sat_. This can be expressed mathematically as follows:

4.3 $$a_{\;f}=\left\{ \begin{array}{cc} 0,\; & u_{\;f}<u_{\mathrm{thr}},\;\\ \frac{u_{\;f}-u_{\mathrm{thr}}}{u_{\mathrm{sat}}-u_{\mathrm{thr}}},\; & u_{\mathrm{thr}}<u_{\;f}<u_{\mathrm{sat}},\;\\ 1,\; & u_{\;f}>u_{\mathrm{sat}}.\; \end{array}\right.$$

The pulse width, **u**_*f*_, is calculated based on a closed-loop feedback controller, described below, and the resultant muscle activation is used to calculate the joint torques of the human model, ***τ***_*h*_ = ***τ***_*n*_ + ***τ***_*f*_, according to OpenSim’s muscle activation dynamics. For this case study, the values used for **u**_thr_ and **u**_sat_ were 100 and 600 μs, respectively, and were kept uniform across the stimulated muscles. The stimulation frequency and amplitude were assumed to be constant.

Both technological agents in this simulation, i.e. the FES electrodes and exoskeleton, are governed by parametrized closed-loop feedback control laws,

4.4 $$\tau_{e}=K_{e}\Delta q+B_{e}\Delta \dot{q}$$

and

**Figure 13.**
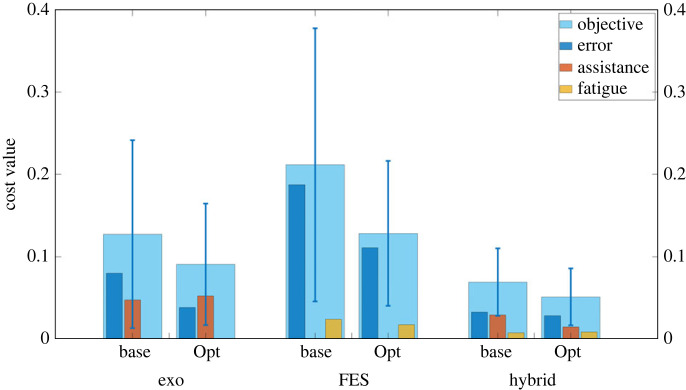
A comparison between three different rehabilitative interventions; exoskeleton-only assistance, FES-only assistance, and hybrid exoskeleton-FES assistance. For each of the three interventions, the estimated tracking error, assistance and muscle fatigue are compared between the case where baseline controller parameters are used and the case where the parameters are optimized for triadic collaboration.

4.5 $$u_{\;f}=K_{\;f}\Delta q,$$

where Δ**q** denotes the measured joint error from the prescribed tracking trajectory. In practice, the gains underpinning each controller are typically manually tuned, which can be a time-intensive process [42]. Using our triadic collaboration framework, coupled with Bayesian optimization as a sampling-based optimizer, we can instead optimize the gains of these controllers to balance the relative impact of fatigue, exoskeleton assistance level and tracking error. The results of such an optimization on the recorded data of 10 healthy individuals are illustrated in figure 13, alongside simulations of dyadic interventions (i.e. using only exoskeleton or only FES assistance).

It can be observed for the exoskeleton-only intervention, the personalized controller gains result in higher assistive forces from the exoskeleton in order to reduce the tracking error and the weighted sum of both, which is effectively a measure of the controller’s ability to provide assistance as needed (box 3). On the other hand, for the FES-only intervention, it can be seen that with the optimized gains for the FES controller both the tracking error and muscle fatigue can be reduced. In either case, we see a measurable benefit in optimizing controller gains for individual subjects.

When the two interventions are combined, an obvious benefit in all three costs can be observed compared with the dyadic scenarios. The tracking error, exoskeleton assistance and muscle fatigue are all significantly reduced compared with the exoskeleton-only intervention and the FES-only intervention cases, regardless of whether the baseline or personalized gains are used. This indicates that due to the triadic collaboration of the three agents, all three costs can be reduced. Similarly as for the dyadic cases, it can be seen that when the personalized gains are used, all three costs are measurably reduced, which is particularly noticeable when compared with the weighted sum of the cost terms. As in case study i, the relative weightings of the respective cost terms could be modified to achieve more specific goals depending on the specific needs of the individual undergoing rehabilitation.

## 5. Discussion

In this concept paper, we have introduced a framework for dealing with problems of dynamic collaboration in multi-agent systems comprising a mixture of humans and robots. The key strengths of our framework are:

— The ability to handle dynamic interaction between multiple human and robotic agents.
— Flexibility to handle various triadic collaboration scenarios via the selection of appropriate ergonomics metrics and task constraints.
— High potential for personalization; modelling constraints can be implemented to represent pathologies such as muscle weakness or injury; and the relative weighting of ergonomics metrics can be changed on an as-needed basis. For example, an assistive exoskeleton for end-of-life care could be driven by a different weight set than rehabilitative care, but otherwise use a similar instantiation of the framework.
— Detailed resolution of ergonomics, enabled via the use of detailed biomechanical models, which in turn enables the consideration of motion health on a deeper level than traditional methods based on kinematics measures.

To illustrate these strengths, we contextualized the framework via two case studies of triadic collaboration: an assisted sit-to-stand transition and hybrid robot/FES-assisted rehabilitation. Notably, each of these tasks was well described by our triadic collaboration framework despite the differences in number of human agents and between outcome measures. In both cases, the benefits of triadic collaboration were evident in that task completion metrics were significantly improved by the addition of a third agent. We see this concretely both in case study i, where the addition of exoskeleton assistance was able to improve the stability of the caree and reduce the physical strain on the carer during the sit-to-stand task, and in case study ii, where the combination of robotic and FES assistance significantly improves rehabilitation outcome measures compared with either intervention individually. Furthermore, the potential to achieve personalized assistance strategies is clear in both cases, and can be achieved via a simple modification of the relative weightings of the objective function. Our case studies also demonstrated the ability to consider detailed biomechanics to a level appropriate for the problem: in case study i, stability and joint loading were the dominant outcome measures, and so a model purely in joint-space was used, while for case study ii muscle-activation dynamics were included to consider the action of the FES assistance. In practice, more or less complicated biomechanical models could be used to represent human agents as appropriate for the problem at hand.

As a source of immediate future work, experiments on healthy human subjects will be carried out to validate the simulation-based results from our case studies and further demonstrate the potential of exoskeletons and robots in multi-agent collaboration scenarios. As part of longer-term research goals, we aim to explore how to optimize the selection of optimization criteria based on the specific motion task. Furthermore, although in this work we have largely focused on the use of detailed biomechanical models to allow for consideration of ergonomics, we aim to explore how other high-level metrics could be employed in triadic collaboration tasks—for example, notions of ethics, or human trust and comfort levels—as part of a human-centred approach [43] to human–robot cooperation.

Throughout our discussion of multi-agent collaborative systems, we have exclusively considered the case of triadic collaboration, involving three agents. However, extensions to cases of more than three agents (i.e. *n-adic collaboration*) can be achieved via the addition of additional constraints and goals as needed, to represent additional agents. In real-world settings, the current state-of-the-art is dyadic collaboration, where humans interact with robots and exoskeletons on a one-to-one basis, which can be considered a special case of the triadic scenarios we have presented here. Our framework offers the potential to extend the current state of the art to larger teams of mixed human and robotic systems, and consequently unlock the associated societal benefit to productivity, ergonomic safety and the well-being of patients and workers.

## Data Availability

Data and relevant code for this research work are stored on GitHub: https://github.com/DanielFNG/ergonomics/tree/royal-society and have been archived within the Zenodo repository: https://doi.org/10.5281/zenodo.7883695 [44].

## Footnotes

1 For example, lower-body investigations may rely on musculoskeletal models which abstract the upper-body geometry in to a single ‘torso’ body.

2 In actuality, the torque limits of the APO are approximately 35 Nm of peak torque output and 15 Nm of sustained torque output [36]. However, we have chosen to artificially increase the APO’s torque limits in simulation so as to allow for evaluation of severely weakened human models.
